# Associated factors, barriers, and interventions to promote physical activity and reduce sedentary time in academics: a systematic review

**DOI:** 10.1186/s12889-025-24092-2

**Published:** 2025-08-13

**Authors:** Fiona Yu, Ritin Fernandez, Sherphard Chidarikire, Lisa Mackay, Melody Smith

**Affiliations:** 1 School of Nursing and Midwifery, College of Health, Medicine and Wellbeing, University of Newcastle, Newcastle, Australia; 2 Centre for Transformative Nursing, Midwifery, and Health Research, A JBI Centre of Excellence, Newcastle, Australia; 3 School of Sport and Recreation, Faculty of Health and Environmental Sciences, Auckland University of Technology, Auckland, New Zealand; 4 School of Nursing, Faculty of Medical and Health Science, University of Auckland, Auckland, New Zealand

**Keywords:** Break, Educator, Employee, Exercise, Faculty, Higher education, Lecturer, Professional teaching fellow, Sedentary, Sit

## Abstract

**Background:**

Low physical activity and prolonged sedentary time harm health and psychological well-being. Being insufficiently active is associated with a risk of numerous non-communicable diseases, including cardiovascular disease, hypertension, and diabetes. Academics comprise a notable portion of the workforce and serve as important role models, yet their positions may involve many sedentary behaviours. The factors associated with their physical activity and sedentary behaviours are unclear. This review aimed to identify the associated factors, barriers, and the effectiveness of interventions in promoting physical activity among academics.

**Methods:**

Systematic review. The data were collated from CINAHL Ultimate, Cochrane, Medline (OVID), Scopus, and SPORTDiscus between March and April 2024. Studies were selected if they (1) involved participants employed in academics, including professional staff (2), investigated the physical activity or sedentary behaviours as a primary outcome and/or the associated factors, and (3) identified the effectiveness of physical activity interventions. Studies with full-text in English were selected if published in peer-reviewed journals before April 2024.

**Results:**

Of the included 46 studies,12 demonstrated 63.10% (95% CI [54.70%, 71.10%], *p* < 0.0001, I^2^ = 96.40%) of participants had low physical activity, while 2 reported a long mean sedentary time per workday of 553.10 min (95% CI [29.51, 1135.72], *p* = 0.70 I^2^ = 0%). Various physical activity measures were utilised, and 42 associated factors were identified, categorised as sociodemographic (*n* = 7), psychological (*n* = 13), environmental (*n* = 6), work-related (*n* = 7), and health-related factors (*n* = 9). Sedentary behaviours were associated with burnout, lower prospective mood, and lower energetic arousal. Barriers to physical activity (*n* = 12) and interventions (*n* = 7) were identified.

**Conclusions:**

The findings suggest that it is imperative to develop institutional strategies and interventions to promote physical activity in the academic population. The diversity and complexity of factors associated with physical activity indicate that further research with a large sample size and a consistent measure is needed to identify the needs of academics for physical activity promotion to enhance well-being.

**Supplementary Information:**

The online version contains supplementary material available at 10.1186/s12889-025-24092-2.

## Background

Physical activity is crucial for maintaining overall health and well-being, reducing stress, and increasing energy levels, thus enhancing productivity, cognitive function, and job satisfaction [1]. A minimum of 150–300 min of moderate-intensity physical activity per week is recommended by the World Health Organisation [2], yet globally, many do not meet this threshold [3]. Physical activity is particularly important for academic employees due to the risk of sedentariness associated with academic roles. Long periods of sedentary time, which comprise most academic duties, lead to poor sleep quality and burnout [4, 5], negatively impacting the quality of life [6]. Prolonged periods of uninterrupted sitting can contribute to numerous other health issues, such as increased blood pressure [7, 8], increased risk of chronic diseases [9], and decreased cognitive function [10]. Regularly interrupting sitting and increasing light-intensity physical activity may help offset the adverse effects of sedentariness [7, 8, 10].

The COVID-19 pandemic has significantly impacted health behaviours and outcomes, including physical activity and sedentary time. Physical activity was significantly reduced on university campuses during the COVID-19 shutdowns, and since then, it has decreased dramatically, as students and lecturers have shifted to more online teaching and learning [11], resulting in more remote classes and less commuting between campuses and their homes. Therefore, identifying barriers to physical activity is key for promoting health within this global context.

Recent literature has identified barriers to physical activity in the general working population across three key areas: work schedules and workplaces, family relationships and household obligations, and an array of barriers spanning individual, economic and ecological factors [12]. Differences also exist in geography; for example, lack of time was the most frequently reported barrier for Australian adults classified as inactive [13]. In Singapore, lack of time, fatigue, pollution, parking, and weather were the main barriers [14], while lack of motivation, energy, and resources were the main barriers for young adults in Malaysia during the COVID-19 lockdown [15]. Overall, barriers to physical activity can vary between countries and populations; therefore, a global perspective of factors associated with physical activity or sedentary behaviour in academics is needed to help develop strategies for promoting physical activity.

Scholars have explored whether physical activity interventions and strategies can help overcome barriers to exercise and improve health outcomes. Chu, Koh [16] identified that physical activity and yoga exercise significantly reduced anxiety and depression in the working population. Sköld, Bayattork [17] found that workplace exercise interventions had a limited impact on improving employees’ mental health and psychosocial environment. Smith, Hosking [18] concluded that improving walkability, quality of parks and playgrounds, and active transport infrastructure could help increase physical activity levels in children and adults. Corbett, Bauman [19] reported that healthy lifestyle interventions (e.g., physical activity) should be encouraged and incorporated into teachers’ daily practice to improve their mental well-being.

Compared to the general population, academic employees typically have higher educational levels and clearer career goals, as measured by metrics such as publications, research grants, and teaching awards [20]. Their strong work focus, encompassing teaching, research, and management, can require long hours and extra workloads, potentially leading to isolation and increased job dissatisfaction [21]. Therefore, promoting physical activity may not only improve physical fitness but also social connections, thus enhancing psychological well-being. Considering the limited research on physical activity among academics, we posed the following questions: What factors are associated with physical activity in academics? What are the barriers to promoting physical activity and reducing sedentary time? What kind of interventions have been conducted with academic staff? Are these interventions effective? In this systematic review, we defined the factors as elements related to physical activity or sedentary behaviours, which could be positive or negative. The academic population and working environment have some unique characteristics compared with office workers in ‘for-profit’ companies (e.g., pay and benefits, autonomy and independence, organisational structure, timetables). As such, interventions should be tailored to the population and working environment. It is helpful to understand the factors associated with physical activity in this population group and interventions that have been successful.

Studying the factors associated with physical activity in academic settings is crucial, as the findings may provide evidence to inform the development of institutional strategies that foster a positive work environment and contribute to a healthier and more productive educational workforce. An enhanced work environment and improved well-being may help employees better cope with challenges, such as a heavy workload, burnout, and prolonged sedentary working hours resulting from staff shortages [22]. In addition, the identified factors may help promote physical activity, which can be beneficial for staff in reducing their stress levels and thus increasing sustainability in the academic workforce. This systematic review aimed to identify the associated factors with physical activity and sedentary behaviour, barriers to promoting physical activity, and types of physical activity interventions and their effectiveness in academics. This review defined the study population in academics as teaching staff members, researchers, and supporting professionals (e.g., administrative workers). The objectives for this review were to (a) identify the physical activity levels and sedentary time in the academic population, (b) determine the associated factors with physical activity, (c) determine the associated factors with sedentary time, (d) identify the barriers to physical activity, and (e) identify the interventions for physical activity promotion and their effectiveness.

## Method

This systematic review was registered in the International Prospective Register of Systematic Reviews (PROSPERO) (CRD42024538723) and reported following the Preferred Reporting Items for Systematic Reviews and Meta-Analyses (PRISMA) protocol.

### Eligibility criteria

We included studies if they: (a) involved participants working in academics (teaching staff members, researchers, and supporting professionals [e.g., administrative workers]), (b) investigated physical activity or sedentary behaviours as a primary outcome and/or the associated factors, and (c) identified the effectiveness of physical activity interventions. It should be noted that in this review, we refer to academics as employees in higher education, working within academia, including lecturers, professors, researchers, and other staff involved in administrative roles. We included peer-reviewed quantitative studies, including experimental and observational articles, published in English with full text before April 2024. The search eligibility criteria are in Supplementary Table 1.

### Search strategy

The search was conducted between March and April 2024 from CINAHL Ultimate, Cochrane, Medline (OVID), Scopus, and SPORTDiscus. Below is an example of search strategies utilised in CINAHL Ultimate:

1. Physical activity OR exercise*OR Break* N5 sit* OR Break* N5 sedentary OR Interrupt* N5 sit*OR Interrupt* N5 sedentary
2. Academi* OR education* OR institution* OR university* OR higher education
3. Lecturer*OR teacher* OR educator* OR researcher* OR professional teaching fellow* or staff* or employee*
4. Facult*

We then used ‘AND’ to combine these four searches to identify the relevant studies. The detailed searches in each database are in Supplementary File 1: Search Strategies.

### Study selection

The search results were exported to EndNote and then to Covidence. Utilising Covidence, we extracted the data by removing duplicates and articles that did not meet the eligibility criteria. Two authors (FY and SC) independently completed the data selection, and the team resolved the disagreement during this process.

### Analysis method

A combination of meta-analysis and narrative synthesis was applied in this systematic review. The JBI SUMARI software was utilised to meta-analyse the prevalence of low physical activity. For the purpose of the meta-analysis, low physical activity was defined per the criteria used in each individual study to maintain consistency with their methodologies and ensure accurate interpretation of the results. The rBiostatistics software (rBiostatistics.com; alpha version) was used to synthesise the sedentary time and quantify the effect sizes from the included studies. Heterogeneity was assessed using the I^2^ statistic, which was classified as low (< 25%), moderate (25%−60%), or high (> 60%) [23]. A p-value (≤ 0.05) was used to determine the significance of the meta-analysis, and a random effect model was followed to synthesise the results. Forest plots were generated to visualise pooled estimates and 95% Confidence intervals.

A descriptive narrative analysis was undertaken to aggregate the findings from studies into key themes. We tabulated findings from individual studies into key categories as sociodemographic, psychological, environmental, work-related, and health-related factors following a theoretical domain framework [24]. All analyses were conducted, ensuring methodological rigour and reproducibility.

### Quality assessment

The quality assessment tool developed by Hawker, Payne [25] was utilised in this systematic review, and studies were graded as good, fair, poor, and very poor. The authors (FY, SC, and MS) independently assessed the studies, and any disagreement was resolved within the team.

## Results

### Study selection

**Fig. 1:**
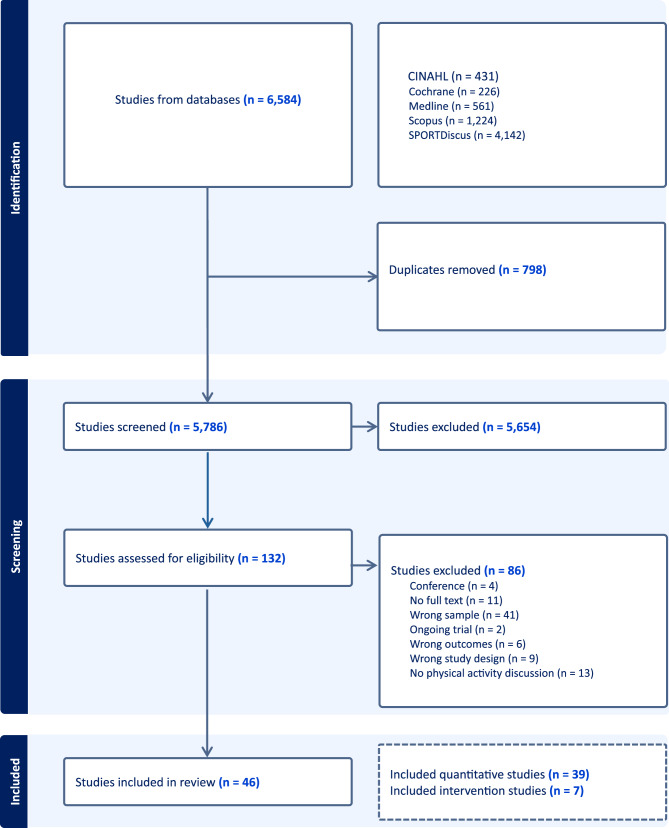
A flow chart for data selection

Of 6,584 studies identified from the five databases, 798 duplicates were removed, leaving 5,786 for title and abstract screening (Fig. 1). After screening the titles and abstracts, we removed 5,654 studies, leaving 132 for full-text review. Of 132 studies, 86 were excluded due to eligibility. These were conference (*n* = 4), no full text (*n* = 11), wrong sample (*n* = 41), ongoing trial (*n* = 2), wrong outcomes (*n* = 6), wrong study design (*n* = 9), and no physical activity discussion (*n* = 13). Finally, 46 remained in the systematic review, including 39 quantitative (Table 1) and 7 intervention (Table 2) studies.

### Study characteristics

The 46 studies were conducted in 20 countries (Supplementary Table 2). Most studies were conducted in the USA (*n* = 13) [11, 26–37] and Turkey (*n* = 5) [4, 6, 38–40]. One study was conducted in Australia and Germany [41]. The remaining were studied in Australia [42], Austria [43], Belgium [44], Brazil [5, 45, 46], Cameroon [47], Canada [48–50], China [51], Ethiopia [52], Indonesia [53], Iran [54–56], Jordan [57], Kenya [58, 59], Malaysia [60], Peru [61], Poland Lopez-Olivares, Slovenia [62], Spain [63, 64], and the UK [65]. Of the 39 quantitative studies, one was designed with a mixed method [31], and the remaining were cross-sectional studies. Of the seven intervention studies, two were randomised controlled trials [37, 42], and five were pre-and post-test studies [35, 36, 44, 50, 65].

A total of 12,301 participants were involved in the 46 studies, with sample sizes ranging from 20 to 1,041 participants (Tables 1 and 2). Seventeen studies did not provide the mean age of the participants, while in the remaining 29 studies, the participants’ mean age (M [SD]) was 32.3 (7.8) years [60] to 50.0 (8.0) years [42]. Participants’ roles spanned academic and professional roles. Academic roles included professors, associate professors, lecturers, and researchers, while the professional roles comprised administrative, clerical, and technical staff. Only five studies specified the faculty from which the participants were (e.g., Faculty of Health Science).

**Table 1:** Summary of the 39 quantitative studies

Author (Ref. number)	Location	Sample characteristics	Sample (*N*)	Physical activity measure	Findings	Quality
Almhdawi 2021 [57]	Jordan	University professors from all faculties	299	International Physical Activity Questionnaire (IPAQ)	Mean physical activity MET (metabolic equivalent): 1167.0 (1914.9). Low physical activity (PA): 58.9%	Fair
Cruz-Ausejo 2023 [61]	Peru	University professors from all faculties	191	IPAQ	Low PA: 93 (48.69%); moderate PA: 42 (21.99%); high PA: 56 (29.32%). Stress was related to high PA and low PA (aPR = 0.19).	Fair
Demuth 2019 [66]	Poland	University professors from all faculties	288	IPAQ	Low PA: 186 (66%); moderate PA: 39 (14%; high PA: 56 (29.32%) Participants with a preference for leisure-time activities in childhood (OR = 283.63) had significantly higher physical activity levels at ages less than 55 years (OR = 0.58 [50–59 yrs]), male gender (OR = 0.49), and non-smoking status (OR = 0.54).	Poor
Diallo 2019 [58]	Kenya	Teaching staff from the College of Health Science	136	Self-developed questions	Regular exercises at least 3 times per week: 61 (44.9%). Physical inactivity was a risk factor associated with low back pain (aOR = 6.0 [never PA]; aOR = 2.8 [rarely PA]; aOR = 1.0 [regular PA]).	Fair
Dias 2017 [45]	Brazil	University professors from the Centre of Human Sciences and Education, Centre of Applied Social and Health Sciences	121	Brief Questionnaire for Measuring Standard Physical Activity in Epidemiological Studies	Inadequate PA: 62 (54.4%). Mean physical activity: 2.61 (1.54) (weekday frequency).	Poor
Fountaine 2014 [26]	USA	University employees categorised as Administration, Faculty, Staff, and Faculties Management	625	Occupational Sitting and Physical Activity Questionnaire (OSPAQ)	Mean sitting time: 350 (158) min/per workday, mean walking time: 59 (55) min/per workday, mean leisure time PA: 3.1 (2.3) days/per week. Sitting time was 69%; walking 12% during a workday. Nearly 75% of a participant’s workday seated.	Fair
Freitas 2020 [5]	Brazil	University professors from all faculties	423	Self-developed	Insufficient time for regular leisure activities was associated with poor sleep quality (PR = 1.52).	Fair
Galof 2021 [62]	Slovenia	Teaching staff in the Faculty of Health Sciences	115	Self-developed questions	Physical activity was associated with back pain (*r* = − 0.57 [leisure PA]; *r* = −0.76 [occupational PA]).	Fair
Giurgiu 2019 [41]	Australia/Germany	University employees	92	Accelerometer	Being sedentary for 30 min without interruption was negatively associated with lower prospective moods and energetic arousal.	Fair
Hariyanto 2023 [53]	Indonesia	Lecturers	355	Global Physical Activity Questionnaire (GPAQ)	Low PA:132 (37.18%); moderate PA:185 (52.11%); vigorous PA: 38 (10.70%).	Fair
Headley 2018 [27]	USA	College employees in Physical Education and Exercise Science	367	OSPAQ; accelerometer	Administrators were more sedentary than faculty members. Sedentary time: 563.1 (96.4) minutes per day.	Fair
Hu 2021 [51]	China	University employees	116	Godin Leisure-Time Exercise Questionnaire (GLTEQ)	Predictors of physical activity were gender (male: β = 12.58), self-reported fitness (β = −0.52), self-efficacy (β = 0.29), and social support from friends (β = 0.70).	Fair
Hudgins 2024 [11]	USA	Faculty staff	38	Monitoring device	The average step count reduced before and after the spring break. Staff’s PA decreased within 30 days and on weekdays due to the COVID-19 lockdown, but maintained a weekend step average.	Fair
Jones 2023 [28]	USA	University employees	103	OSPAQ	Mean sitting time: 362.39 (108.75) min/per day. Age, sex, employee status, and work engagement not associated with occupational sitting times.	Fair
Khubchandani 2009 [29]	USA	University employees	415	Self-developed questions	Participants with high stress were less likely to exercise (χ² = 19.19). The barriers to exercise on campus included time constraints, work assignments, the cost of gym membership, lack of parking, and distance from the exercise facility.	Fair
Kirk 2012 [48]	Canada	Assistant professors	267	GLTEQ	Compared to participants who remained active, those who were inactive considered PA a hassle or inconvenience to exercise (F = 11.59). Participants who were active in PA had a better intention (F = 36.63), perceived behavioural control (F = 9.70), affective attitude (F = 0.05), and descriptive norm (F = 5.24), and were more likely to take time away from obligations (F = 5.24). They also had better control over their limited free time (F = 5.46), inconsistent schedule (F = 10.08), and heavy work demands (F = 6.83). Active participants had greater physical fitness than inactive participants (F = 3.49).	Fair
Kwiecień‑Jaguś 2021 [67]	Poland	University employees, including professors, associate professors, and lecturers from Health Science, Medicine, and Pharmacy and the Intercollegiate Faculty of Biotechnology	276	IPAQ	Low PA: 222 (80.43%). Low PA was associated with alcohol consumption (OR = 3.85) and general assessment of health (OR = 7.36). PA was not correlated with self-efficacy, age, marital status, or the number of children.	Fair
Leininger 2015 [30]	USA	University employees	308	IPAQ	Met the recommendations for weekly PA: 52.3% Barriers included time constraints, using one’s program, living too far, scheduling conflicts, being unaware of the program, not being interested in offerings, and not being on campus often.	Fair
Lopez-Olivares 2021 [63]	Spain	Professors	127	IPAQ	No significant differences between men and women regarding PA levels. The level of PA was associated with a Western diet pattern but not a Mediterranean one.	Fair
Mohammadi 2016 [56]	Iran	University employees	379	A Questionnaire Physical Discomfort of Staff (ACSM)	Barriers included lack of motivation, family obligations, limited time, discouraging exercise, and lack of attention to workplace sports culture.	Poor
Mohan 2015 [60]	Malaysia	University employees from Health Sciences, Information Technology, Hotel Management, Business Management, Pharmacy, and the Centre for Foundation Studies	228	Dutch Musculoskeletal Questionnaire (DMQ)	Stood for long periods: 64.1%, sat for long periods: 78.9%, and walked for long periods: 36.8%. Physical risk factors not related to work-related musculoskeletal disorders.	Fair
Motevalli 2023 [43]	Austria	Academic staff	1,041	Self-developed questions	Decrease in PA level: 37.5%; increase in PA level: 27.9% Living area (urban or rural; χ² = 13.99) and Austrian region (χ² = 11.86) were significant indicators of the direction of PA changes among academic staff.	Fair
Moueleu Ngalagou 2019 [47]	Cameroon	Teaching staff members	303	Ricci-Gagnon questionnaire	Sport and physical activities showed a significant protective effect against burnout (OR = 2.07) and less time for leisure (OR = 2.23). Sedentariness (physical inactivity) was a risk factor associated with burnout.	Fair
Omondi 2007 [59]	Kenya	Lecturers	120	Self-developed questions	Participants had light activities (lecturing [standing], driving, and walking) and moderate activities (climbing stairs, walking briskly, and cycling), but no one participated in high-intensity PA. Physical activity patterns were significantly related to the overall health (χ² = 27.54).	Poor
ÖZcan 2021 [6]	Turkey	Physical education teachers	155	Godin-Shephard Leisure-Time Physical Activity Questionnaire (GSLTPAQ)	A negatively significant but weak correlation between leisure-time PA and the quality of life during COVID-19 (*r* = −0.22).	Poor
Özdinç 2019 [38]	Turkey	Academicians consisting of lecturers, research assistants, instructors, assistant professors, associated professors, and professors	142	International Physical Activity Questionnaire Short Form (IPAQ-SF)	Mean PA level: 1067.31(1866.95) MET-minute/week. Daily sitting time: 7.55 (4.19) hours/day. Low PA: 59 (55.1%). PA was one of the factors affecting academic musculoskeletal problems.	Fair
Pérussee-Lachance 2010 [49]	Canada	Staff members	653	Self-developed questions	Less than 150 min/week of moderate-intensity PA: 65.5% of females and 60.7% of males. This was a factor associated with obesity.	Poor
Pirincci 2008 [39]	Turkey	Academic staff	509	Health Promotion Lifestyle Profile (HPLP)	Mean exercise level: 2.05 (0.76). Exercise was one of the factors in health-promoting lifestyle behaviours. Participants with healthy profiles exhibited higher PA levels compared to those with non-healthy profiles (F = 4.42).	Poor
Redondo-Flórez 2020 [64]	Spain	University professors	470	Self-developed questions	Males showed significantly higher values in the weekly team sport exercises (F = 5.574).	Fair
Schmelling 1985 [31]	USA	University employees	135	Self-developed questions	Regular exercise improved well-being, heart and lung health, energy, and strength. Exercises included brisk walking, jogging, callisthenics, aerobic dancing, bicycling, and swimming. Exercise was correlated with a positive attitude (*r* = 0.77) and intention (*r* = 0.65).	Poor
Shahlaee 2022 [56]	Iran	Faculty members	284	IPAQ	lacked physical activity:72%; physical activities during the pandemic: 28%; strenuous or moderate physical activity during the coronavirus 0%. Moderate PA (t = −17.32) and vigorous PA (t = −27.74).	Poor
Soares 2019 [46]	Brazil	Professors	222	Self-develop questions	Physical activity was associated with stress (OR = −0.40).	Fair
Sobhanian 2020 [54]	Iran	Faculty members of the Medical Science	244	The short questionnaire for the measurement of habitual physical activity in epidemiological studies	Mean physical activity: 2.88 (0.61). There was no significant statistical relationship between physical activity and general health (physical symptoms, anxiety, social function, and depression).	Poor
Terzano 2011 [32]	USA	University employees	111	Self-developed questions	Transit mode was related to recreational physical activity. People walking or biking to campus engaged in more recreational physical activity than using a car or mass transit (F = 12.77).	Poor
Whipple 2008 [33]	USA	University employees	653	Self-developed questions	Regarding the barriers to physical activity participation, compared to non-maintainers, maintainers had less negative affect (F = 114.18), a more positive attitude (F = 8.29), higher exercise self-efficacy (F = 84.69), higher confidence (F = 106.65), less resistance to exercises (F = 39.99), and more continuously exercise in the bad weather (F = 75.84).	Fair
Wilkerson 2019 [34]	USA	University employees consisting of faculty, professional staff, and office, clerical and technical staff	502	OSPAQ	Mean standing time: 72.49 (73.48) min/day. Standing time was associated with sex (men standing longer; t = 3.50 [1, 442]), education (participants with advanced degrees standing longer; H [2] = 20.11), and occupation (faculty standing longer; H [2] = 52.94).	Poor
Yildiz 2023 [4]	Turkey	Research assistants, lecturers, assistant professors, associate professors, and professors	214	IPAQ	Significant and strong negative relationships between PA and burnout. Significant positive relationships between PA and job satisfaction and quality of life. Office tenure, age, and computer usage time (*r* = − 0.76) were correlated with PA.	Fair
Yorulmaz 2022 [40]	Turkey	Academicians	381	Self-developed questions	No regular exercise: 81%. An increase in active time affected the musculoskeletal system’s pain intensity (β = −0.10).	Fair
Zenbaba 2022 [52]	Ethiopia	Academic staff	416	Self-developed questions	Frequently perform physical exercise per week: 34.9%; 5–9 h daily sitting: 52%; 10 h or above sitting: 7.7%. Engaging in physical activity was associated with work-related musculoskeletal symptoms (AOR = 3.32).	Fair

The reference numbers of the included studies are listed in Supplemental File 2: Referencing

**Table 2:** Summary of the seven intervention studies

Author Year (Reference)	Location	Design	Sample characteristics Sample size	PA measure	Intervention Conduction time	Key findings	Quality
Brett 2017 [65]	UK	Pre-/post-test	Administrative and academic staff *N* = 20	Pedometer	Recording participants’ daily walking steps 8 weeks	A mean increase of 385.66 (310.74) steps per day relative to baseline.	Fair
Brinthaupt 2010 [35]	USA	Pre-/post-test	University faculty and staff *N* = 58	Fitness test and psycho-behavioural inventory	A 3-hour orientation followed by fitness coaching 10 weeks	A significant increase in physical fitness, health, and happiness; a significant decrease in exercise barriers (e.g., insufficient time, lack of knowledge and confidence).	Fair
Dawson 2008 [50]	Canada	Pre-/post-test	University faculty and staff *N* = 194	Exercise and barriers to self-efficacy	Monitoring sedentary behaviours, identifying barriers, and setting up goals 10 weeks	The group-based intervention significantly increased confidence to exercise and overcome barriers compared to the Internet and no-intervention groups	Fair
Haines 2007 [36]	USA	Pre-/post-test	College faculty and staff *N* = 60	Pedometer	A computer-based educational program (pedometer, nutrition, and stress reduction) 12 weeks	The program moderately affected fitness, mood, health awareness, nutrition, health, anxiety, happiness, weight loss, productivity, and absenteeism.	Fair
Higham 2023 [42]	Australia	Randomised controlled trial (RCT)	Academics *N* = 50	Godin Leisure-Time Exercise Questionnaire (GLTEQ)	Supervised training at an onsite facility 3 times (60 min each) weekly. The training consisted of resistance and aerobic exercises. No training for the control group. 14 weeks	There was a significant increase in total weekly leisure activity scores between the intervention group (*n* = 23) and the control group (*n* = 27): before and after the intervention (time by group: *p* = 0.024, effect size = 0.326).	Fair
Howie 2021 [37]	USA	RCT	University employees *N* = 29	Actigraph GT9x accelerometers International Physical Activity Questionnaire (IPAQ)	Walking 30 min, 5 days per week for 4 weeks. The in-person group (supported); the virtual group (no interactions). 4 weeks	There was a significant group-by-time interaction for PA, with a greater increase in the virtual group (*n* = 13) compared to the in-person group (*n* = 16).	Fair
Opdenacker 2008 [44]	Belgium	Pre-/post-test	University employees consisting of professors, academic assistants, and technical assistants *N* = 66	IPAQ	A coaching program in person for the face-to-face group (*n* = 33) and by phone for the telephone-based group (*n* = 33). 12 weeks	Both groups significantly increased their leisure-time PA and decreased their sitting time from the pretest to the post-test. No significant changes over time or between groups for work, garden or total PA.	Fair

The reference numbers of the included studies are listed in Supplemental File 2: Referencing

Sixteen types of physical activity measures were used in the included studies (Supplementary Table 3). Most studies used the International Physical Activity Questionnaire (*n* = 10), accelerometers (*n* = 6), Occupational Sitting and Physical Activity Questionnaire (*n* = 4), and Godin Leisure-Time Exercise Questionnaire (*n* = 3). Fourteen studies self-developed questionnaires to measure physical activity, which differed from study to study, increasing the variety of the measures. Two studies applied subjective and objective measures to assess participants’ physical activity levels and sedentary behaviours [27, 37].

### Risk of bias in studies

Table 3 shows the quality assessment. A total of 29 studies had insufficient or poor discussion of implications and usefulness, and 22 briefly mentioned ethics approval, did not discuss ethical issues, or did not demonstrate the awareness of researchers’ bias. A total of 13 studies had minimal or no description of their findings’ generalisability, 8 did not provide sufficient information on data analysis, and 4 lacked sampling strategies. In addition, 4 had inadequate backgrounds, 2 were missing details in the description of the methods, and 1 missed an abstract. All studies were retained; however, their quality assessment scores were taken into account when considering their relative contribution to the narrative analyses.

**Table 3:** Summary of the identified areas of risk of bias in the included studies

Risk of bias	1	2	3	4	5	6	7	8
Author year (Reference)								
Almhdawi 2021 [57]						✓	✓	✓
Brett 2017 [65]						✓		
Brinthaupt 2010 [35]					✓	✓		
Cruz-Ausejo 2023 [61]								✓
Dawson 2008 [50]						✓		✓
Demuth 2019 [66]		✓				✓	✓	✓
Diallo 2019 [58]								✓
Dias 2017 [45]					✓		✓	✓
Fountaine 2014 [26]								✓
Galof 2021 [62]						✓		
Haines 2007 [36]					✓	✓		
Hariyanto 2023 [53]						✓		✓
Higham 2023 [42]								✓
Howie 2021 [37]		✓				✓		✓
Hu 2021 [51]				✓				✓
Hudgins 2024 [11]				✓		✓		✓
Jones 2023 [28]						✓		✓
Kirk 2012 [48]								✓
Kwiecień‑Jaguś 2021 [67]								✓
Lopez-Olivares 2021 [63]								✓
Mohammadi 2016 [56]			✓		✓	✓	✓	✓
Moueleu Ngalagou 2019 [47]							✓	✓
Omondi 2007 [59]					✓	✓	✓	✓
ÖZcan 2021 [6]						✓	✓	✓
Özdinç 2019 [38]						✓		✓
Pérussee-Lachance 2010 [49]			✓			✓		✓
Pirincci 2008 [39]	✓	✓				✓	✓	✓
Schmelling 1985 [31]					✓	✓	✓	
Shahlaee 2022 [54]				✓		✓	✓	✓
Soares 2019 [46]							✓	
Sobhanian 2020 [55]				✓			✓	✓
Terzano 2011 [32]					✓	✓	✓	✓
Whipple 2008 [33]						✓		✓
Wilkerson 2019 [34]		✓				✓		✓
Zenbaba 2022 [52]					✓			✓

A tick ✓ indicates identified areas of risk of bias in the studies

The reference numbers of the included studies are listed in Supplemental File 2: Referencing

The columns listed below show the risk of bias areas that we identified

Column 1. Unclear title and abstract

Column 2. Missing information in the background

Column 3. Unreliable tool utilised in the method

Column 4. Insufficient description of the sampling strategy

Column 5. Insufficient or no explanation of data analysis

Column 6. Poor or insufficient discussion of ethics and bias

Column 7. Insufficient or no discussion of transferability or generalisability

Column 8. Insufficient or poor discussion of implications and usefulness

### Physical activity and sedentary behaviour

#### Physical activity

**Fig. 2:**
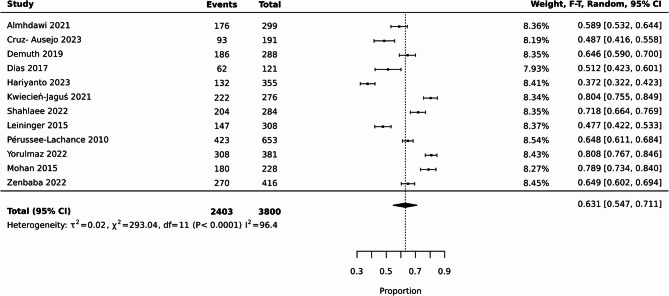
A meta-analysis of low physical activity from 12 studies

Pooled results from 12 studies demonstrated that 63.10% (95% CI [54.70%, *p* < 0.0001, 71.10%], I^2^ = 96.40%) of participants had low physical activity (Fig. 2). It is worth noting that we were unable to include other studies in this meta-analysis due to missing information in their physical activity reports. These 12 studies utilised subjective measures for physical activity levels [30, 40, 45, 49, 52–54, 57, 60, 61, 66, 67].

#### Sedentary time

Pooled data from two studies [26, 27] for the mean sedentary time per workday was 553.10 min (95% CI [29.51, 1135.72], *p* = 0.70 I^2^ = 0%) (Supplementary Fig. 1).

### Factors associated with physical activity

From the 39 quantitative studies, 42 factors associated with physical activity were identified and categorised as sociodemographic (*n* = 7), psychological (*n* = 13), environmental (*n* = 6), work-related (*n* = 7), and health-related factors (*n* = 9) (Supplementary Table 4).

#### Sociodemographic factors

Seven factors associated with physical activity were ascertained within the sociodemographic category: age, sex, education level, occupation, job grade, smoking, and alcohol consumption. One study identified that participants’ physical activity levels decreased when they were older and reduced by 50% for those above 59 years old (OR = 0.66 [up to 39 yrs], OR = 0.59 [40–49 yrs], OR = 0.58 [50–59], *p* ≤ 0.01) [66]. Four studies found sex associated with physical activity and reported that males were more active than females (OR = 0.49, *P* ≤ 0.01; β = 12.58, 95% CI [2.75, 22.41], *p* < 0.05; F = 5.574, *p* = 0.019; t [1,442] = 3.5, *p* < 0.001) [34, 51, 64, 66]. Wilkerson, Usdan [34] found that participants with an advanced degree stood longer than those with a bachelor’s degree (H [2] = 20.11, *p* < 0.001) and that faculty members stood longer or were more active than clerical and administrative staff (H [2] = 52.94, *p* < 0.001). Yildiz and Kocaman [4] reported that the higher the academic title was, the lower the physical activity levels were (*p* = 0.006). Demuth and Czerniak [66] found that smokers had less physical activity than non-smokers (OR = 0.54, *p* ≤ 0.01), while Kwiecień-Jaguś, Mędrzycka-Dąbrowska [67] identified that participants with high amounts of alcohol consumption had a low level of physical activity (OR = 3.85, 95% CI [1.14, 13.02], *P* = 0.029).

#### Psychological factors

Thirteen psychological factors were associated with physical activity, including four negative and nine positive factors. They were stress, negative affect (e.g., feeling depressed), a hassle/inconvenience to exercise, resistance to exercise, exercise self-efficacy, exercise social support, positive attitude, confidence, intention, perceived behaviour control, taking time from obligations, and descriptive norm (e.g., valuing other people’s opinions). Three studies identified that stress was negatively associated with physical activity (aPR = 0.19, 95% CI [0.06, 0.59], *p* = 0.004; χ² = 19.19, *p* < 0.001; OR = −0.40, 95% CI [−0.51, −0.29], *P* = 0.01) [29, 46, 61]. Whipple, Kinney [33] found that negative affect (F = 114.18, *P* < 0.001) and resistance to exercise (F = 39.99, *p* < 0.001) were negatively related to physical activity, while Kirk and Rhodes [48] reported that exercise being reported as a hassle/inconvenience was negatively associated with physical activity (F = 11.59, η² = 0.10, *p* < 0.001).

For the positive factors, two studies identified that exercise self-efficacy was positively associated with physical activity (β = 0.29, 95% CI [0.14, 0.43], *P* < 0.01; F = 84.69, *p* < 0.001) [33, 51]. Hu, Hu [51] reported that exercise social support was also positively related to physical activity (β = 0.70, 95% CI [0.19, 1.21], *P* < 0.01), while Yildiz and Kocaman [4] found that job satisfaction was associated with physical activity (*p* < 0.05). Three studies identified that a positive attitude towards physical activity was related to physical activity (F = 0.05, *p* < 0.001; *r* = 0.77, *p* < 0.001; F = 8.29, *p* < 0.01) [31, 33, 48]. One study reported that participants with confidence to exercise had high physical activity (F = 106.65, *p* < 0.001) [33], while two studies found that participants with an intention to be physically active increased their physical activity levels (F = 36.63, *p* < 0.001; *r* = 0.65, *p* < 0.01) [31, 48]. Kirk and Rhodes [48] also found that perceived behaviour control (F = 9.70, *p* < 0.001), taking time from obligations (F = 5.83, *p* < 0.01), and descriptive norms (e.g., valuing other people’s opinions) (F = 5.24, η² = 0.05, *p* < 0.01) were positively associated with physical activity.

#### Environmental factors

Six environmental factors were associated with physical activity: geographic region, urban or rural areas, transit mode for commuting to work, inconvenience to exercise, weather, and COVID-19 lockdown. Specifically, Motevalli, Drenowatz [43] reported that physical activity was related to the geographic regions (χ² = 11.855, *p* = 0.018) (e.g., lower in eastern Austria compared to centre and western Austria) and was higher in those living in rural areas compared to urban areas (χ² = 13.966, *p* < 0.001). Two studies identified that the COVID-19 lockdown was associated with physical activity [11, 54]. For example, Shahlaee and Nasiri [54] described that the participants reduced their moderate physical activity (t = −17.32, 95% CI [−0.95, −0.84], *p* = 0.003) and vigorous physical activity (t = −27.74, 95% CI [−1.23, −1.15], *p* = 0.001) due to the COVID-19 lockdown. Hudgins, Kurti [11] also found a decrease in physical activity in a university community after the COVID-19 lockdown (*p* < 0.05). Whipple, Kinney [33] reported that environmental inconveniences (e.g., not enough facilities) (F = 49.26, *p* < 0.001) and weather (F = 75.84, *p* < 0.001) affected physical activity, while Terzano and Morckel [32] identified that using active modes for commuting to work was a factor associated with physical activity (F = 12.77, *p* < 0.01).

#### Work-related factors

Seven work-related factors negatively associated with physical activity were computer usage time, lack of free time, inconsistent schedule, heavy work demands, burnout, and work-related musculoskeletal symptoms. Specifically, Yildiz and Kocaman [4] reported that the more time spent on computer usage, the less physical activity was engaged (*r* = −0.76, *p* = 0.03). Kirk and Rhodes [48] identified that declined physical activity was related to lack of free time (F = 5.46, η² = 0.05, *p* < 0.01), inconsistent schedule (F = 10.08, η² = 0.09, *p* < 0.001), and heavy work demands (F = 6.83, η² = 0.06, *p* < 0.01). Two studies found burnout negatively associated with physical activity (OR = 2.07, 95% CI [1.27, 3.38], *p* = 0.04; *p* < 0.05) [4, 47]. Moueleu Ngalagou, Assomo-Ndemba [47] identified that less time for leisure was a factor negatively associated with physical activity (OR = 2.23, 95% CI [4.02, 13.00], *p* = 0.016), while Zenbaba, Sahiledengle [52] reported that work-related musculoskeletal symptoms caused a decrease in physical activity (AOR = 3.32, 95% CI [1.43, 7.74, *p* = 0.009).

#### Health-related factors

Nine health-related factors associated with physical activity: pain, quality of life, Western diet pattern, physical fitness, promoting lifestyle behaviours, poor sleep quality, ways of spending leisure time in the past, health status, and the effect of COVID-19 on quality of life. Four studies explored the relationship between pain and physical activity [38, 40, 58, 62]. Diallo, Mweu [58] determined that knee pain was a factor in reducing physical activity in a lifetime (*p* = 0.033), while Yorulmaz, Karadeniz [40] found that an increase in physical activity time reduced the intensity of musculoskeletal pain (β = −0.103, t = −2.204, 95% CI [−8.474, −0.483], *p* = 0.028). Diallo, Mweu [58] reported that regular exercise strengthened lower back muscles, thus reducing back pain (*p* = 0.031), while Galof and Šuc [62] identified that physical activity at leisure time (*r* = −0.574, *p* < 0.001) and at work (*r* = −0.758, *p* < 0.01) reduced back pain.

Yildiz and Kocaman [4] explained that quality of life was positively correlated with physical activity (*p* = 0.010), while López-olivares, De Teresa Galván [63] ascertained that participants who adhered to a Western diet pattern had a decrease in physical activity (*p* = 0.010). In the study by Hu, Hu [51], participants with low scores of self-reported fitness (a low score indicates higher physical fitness) had high levels of physical activity (β = −0.52, 95% CI [−0.83, −0.21], *p* < 0.01), while Kirk and Rhodes [48] reported that physical activity improved fitness (F = 3.49, η² = 0.03, *p* < 0.05), and promoting lifestyle behaviours increased physical activity (F = 4.42, *p* = 0.010) [39].

Poor sleep quality was associated with insufficient exercise (PR = 1.52, 95% CI [1.23, 1.86], *p* < 0.001) [5], and participants who preferred to be active (e.g., active or passive forms of leisure) in their childhood and teenage years had higher physical activity than those who preferred passive forms of leisure (β = 5.61, OR = 283.63, *p* < 0.01) [66]. Participants with better health status were more engaged in physical activity (χ² = 27.54, *p* < 0.05; OR = 0.736, 95% CI [1.72, 31.52], *p* = 0.007) [59, 67], and physical activity reduced the effect of COVID-19 on quality of life (*r* = −0.2.2, *p* < 0.01) [6].

### Factors associated with sedentary time

Four studies investigated the associated factors with sedentary behaviours. Moueleu Ngalagou, Assomo-Ndemba [47] identified that sedentariness was significantly associated with burnout (*p* = 0.007), while Giurgiu, Koch [41] found that high sedentariness was associated with lower prospective mood (standardised BC = −0.082, *p* ≤ 0.001) and lower energetic arousal (standardised BC = −0.019, *p* < 0.001). Jones, Credeur [28] found that sedentariness was not related to age, sex, employee status, or work engagement, while Mohan, Justine [60] identified no association between sedentary time and work-related musculoskeletal disorders.

### Barriers to physical activity

Three studies explored the barriers to physical activity and identified time constraints as the biggest barrier [29, 30, 56] (Supplementary Table 5). Distance to exercise facility [29, 30] and financial cost [29, 56] were other barriers to physical activity. In addition, work assignments [29], scheduling conflicts [30], resistance to using offered exercise programmes [30], and family responsibilities [56] were also identified barriers to exercise. Moreover, lack of parking [29], lack of motivation [56], lack of attention to workplace sports culture [56], unawareness of exercise programmes [30], and no interest in participating in exercise promotion [30] were identified as barriers to attending physical activities.

### Interventions

The interventions in the seven studies included an 8-week recording of participants’ daily steps, a 10-week fitness coaching programme, a 10-week sedentary monitoring programme, a 12-week educational programme, a 14-week resistance and aerobic exercise programme, a 4-week walking programme, and a 12-week exercise coaching programme (Table 2).

These seven studies identified that the programmes significantly improved participants’ physical activity compared to baseline after 4–14 weeks. Specifically, three studies found that the interventions increased the daily steps [65] and physical fitness as a consequence of a decrease in body fat (F [1, 57] = 35.47, *p* < 0.001) [35], and BMI (*p* = 0.024) [36]. Another three [37, 42, 44] identified that participants increased their leisure time physical activity scores after the interventions (*p* = 0.024, effect size = 0.326) (*p* = 0.0498) (F = 6.904, *p* < 0.05). Two [35, 50] reported that the interventions helped participants increase their exercise self-efficacy (F [2, 66] = 3.82, *p* = 0.05, η^2^ = 0.08) in overcoming barriers to exercising, such as not enough time for exercise (F [1, 57] = 8.10, *p* = 0.006), lack of information/knowledge (F [1, 57] = 17.20, *p* < 0.001), lack of confidence (F [1, 57] = 15.17, *p* < 0.001), too intimidated to exercise (F [1, 57] = 7.64, *p* = 0.008), no exercise partner (F [1, 57] = 9.12, *p* = 0.004), history of giving up (F [1, 57] = 7.13, *p* = 0.010), and fear of injury (F [1, 57] = 4.60, *p* = 0.036).

## Discussion

This systematic review aimed to identify factors associated with physical activity levels, sedentary time, and related barriers and interventions in academic settings. A total of 46 studies were included in the review. Following a theoretical domain framework [24], the 42 factors associated with physical activity were categorised into five groups: sociodemographic, psychological, environmental, work-related, and health-related factors. In addition, factors associated with sedentary behaviours, barriers to physical activity, and physical activity intervention programmes were also identified. This is the first systematic review in the academic field that combines meta-analysis and narrative synthesis to examine physical activity and sedentary behaviours.

This review identified low or insufficient physical activity levels and prolonged sedentary time in academics. These findings align with a global report, which states that more than one in four adults do not meet the recommended physical activity levels [3]. The findings indicate that it is necessary to develop interventions for physical activity promotion to enhance well-being among people working in academia.

The review found that physical activity was associated with age, sex, education level, job grade, smoking, and alcohol consumption. Low physical activity levels were identified in the following groups: people aged 59 years or above, females, individuals with higher academic titles, smokers, or those who consumed higher amounts of alcohol. Therefore, policymaking, education campaigns, community programmes, lifestyle interventions, and financial support to reduce physical activity inequalities should be developed to meet the needs of these diverse university employees. This finding aligns with the global report that provides different recommendations for reaching physical activity targets according to sociodemographic characteristics [3]. It also reflects that gender differences should be incorporated into support or educational programmes [68]. For example, a thoughtful approach that considers the differences in characteristics between males and females may need to be utilised when designing a physical activity program to encourage women to overcome the barriers and increase their motivation for exercise. Further research is needed to explore the associations between physical activity and other sociodemographic factors to develop specific institutional strategies to tailor people’s well-being needs.

Thirteen psychological factors were identified influencing physical activity. The positive factors were exercise self-efficacy, exercise social support, job satisfaction, positive attitude, confidence, intention, perceived behaviour control, taking time from obligations, and descriptive norm (e.g., valuing other people’s opinions). The negative psychological factors entailed stress, negative affect (e.g., feeling depressed), a hassle or inconvenience to exercise, and resistance to exercise. This finding suggests that psychological factors play a significant role in determining whether academic staff engage in physical activity. For example, some people may utilise exercise as an approach to reducing their stress and symptoms of depression to promote a sense of calm [29, 41]. Conversely, others may avoid exercise when they feel stressed or depressed [46, 61], viewing it as burdensome [48]. Interventions that consider these psychological factors, such as stress-reduction strategies and confidence-building exercises, could be effective in increasing physical activity participation among academics. It is also crucial to recognise how time constraints and a lack of motivation can hinder regular exercise [33, 48].

Six environmental factors influencing physical activity were identified: geographic region, urban or rural areas, the transit mode used for commuting to work, the inconvenience of exercising, the weather, and the COVID-19 lockdown. These findings highlight the complex relationship between physical activity and environmental factors. For example, promoting cycling or walking to work could be facilitated by creating a safer and more accessible environment [18, 69]. Additionally, universities could foster a positive work culture by encouraging fitness and investing in on-campus exercise facilities [70, 71]. Further research is needed to understand how the environmental factors can promote physical activities and reduce sedentary behaviours in academic settings.

Seven work-related factors were associated with physical activity: computer usage time, lack of free time, inconsistent schedule, heavy work demands, burnout, less time for leisure, and work-related musculoskeletal symptoms. This finding reveals the barriers to physical activity participation due to the sedentary nature of academic work (e.g., significant job demands and sitting for long hours). Aligning with these work-related factors, we identified that the main barriers were time constraints, work assignments, and schedule conflicts [29, 30, 56]. To address these issues, multicomponent interventions, including policy changes, supporting flexibility in work schedules [72], workstation assessments and adaptations (e.g., installation of standing desks) [73], and programmatic interventions (e.g., computer prompt programs for activity breaks) [74] are necessary to facilitate a more active work environment.

Nine health-related factors associated with physical activity were identified: pain, quality of life, Western diet pattern, physical fitness, promoting lifestyle behaviours, poor sleep quality, ways of spending leisure time in the past, health status, and the effect of COVID-19 on quality of life. This finding indicates that people with high physical activity may have a better quality of life, healthy lifestyle, and better sleep quality [4, 5, 39]. A previous review evidenced this [75], which found that professors with low physical activity and sedentary lifestyles were at high risk of cardiovascular, dyslipidaemias, and metabolic diseases. These findings reinforce the importance of promoting physical activity as part of a broader health and wellness strategy for academic staff.

Three factors associated with sedentary behaviours were identified: burnout, lower prospective moods, and lower energetic arousal. Burnout is associated with poor work conditions (e.g., high job demands and working long hours) [47], thus resulting in less or no time for exercise, which negatively impacts psychological well-being (e.g., mood) [41, 70]. This suggests that addressing burnout should be a priority for universities aiming to promote physical activity [76]. Therefore, it is necessary to develop resilience training programmes that may help prevent burnout, thus promoting physical activity [77, 78]. No demographic factors were identified as associated with sedentariness in this review, but a previous review reported that age, body mass index, and socioeconomic status were important factors to consider in the working population [70].

Aligning with the studies [12–15], this review identified time constraints as the main barrier to physical activity in academics. Distance to the exercise facility, financial cost, work assignments, schedule conflicts, and family responsibilities were also key barriers. Additionally, lack of parking, low motivation, resistance to using offered exercise programmes, lack of attention to workplace sports culture, unawareness of exercise programmes, and no interest in participating in exercise promotion were barriers to physical activity. Low physical activity may not necessarily indicate a lack of interest or awareness but rather reflect the challenge of balancing multiple competing priorities. Individuals juggling family responsibilities, career advancement, and research commitments may consciously deprioritise physical activity in favour of immediate professional and personal obligations. In such cases, the issue is not a lack of motivation but rather a strategic decision based on time constraints and perceived priorities. This review was not designed to determine what priority people placed on physical activity versus other activities, but instead, to report on the barriers identified in included studies. Understanding physical activity within this broader context highlights the need for integrated approaches that emphasise how physical activity can support, rather than compete with, professional and personal goals. These findings highlight the need for comprehensive interventions that address these multiple barriers, such as flexible work schedules, subsidised gym memberships, and increased awareness of available exercise options.

Lastly, this review identified the effectiveness of physical activity interventions, including recording or monitoring sedentary behaviours, fitness coaching, nutrition and stress reduction educational programs, and resistance and aerobic exercise. These interventions were shown to improve participants’ physical fitness, reduce sitting time, and enhance social interactions, contributing to increased happiness and overall well-being [35, 36, 44, 50]. These findings align with previous studies [19, 79] that highlight the importance of structured interventions to reduce sedentary time and promote physical activity among academic staff.

The overall findings further underscore the need to promote physical activity in academic work settings to reduce employees’ social, professional, and physical isolation by boosting their motivation to be active, reducing sedentary time, and encouraging participation in physical activity programs. The multiple factors identified in this systematic review indicate the complexity of promoting physical activity in academic settings, suggesting that reducing sedentary time is a collaborative effort among individuals, workplaces, and organisations, rather than just individual behaviours [80]. Therefore, a healthy academic workplace can be developed by taking these identified factors and barriers into consideration and providing health education, which may have a powerful impact on academic staff’s physical activity and sedentary behaviours.

A major strength of this review is that the application of the theoretical domains framework [24] facilitates an ecological perspective, highlighting the array of factors that can impact physical activity from individual-level factors (including competing priorities) to institutional and policy supports. A key strength of using an ecological approach in workplace physical activity research is the focus on the important role of the workplace environment in supporting physical activity at the institutional level but also through mitigating individual barriers to physical activity (e.g., allowing for glide time, considering workloads and scheduling, and subsidising individual and family physical activity programmes). Indeed, in the intervention studies identified, we found evidence for the efficacy of workplace programmes in reducing individual-level barriers to physical activity.

### Limitations

There were several limitations in this systematic review. Firstly, the studies included were mostly cross-sectional, meaning that causality could not be determined. The heterogeneity of measures made it impossible to meta-analyse the study findings to determine the effect size for the seven intervention studies. The lack of standardised definitions of low physical activity, inconsistent physical activity measures and missing information further complicated data synthesis. Additionally, non-English publications and qualitative studies were not included, which may have excluded some insightful information from the findings.

### Implications for research and practice

Several interventions could be applied to support people in academic settings. Creating policies that support work-life balance, provision of well-being programs, application of new technologies, and workshops may help promote physical activity [81]. Setting up onsite facilities and active workspaces, developing outdoor green spaces, initiating programmes to facilitate active travel modes or subsidising gym membership fees may promote physical activity and reduce sedentary behaviours [18, 82]. Cognitive behavioural therapy with specific goal setting may help reinforce individuals’ motivation, confidence, and self-esteem to participate in physical activity [83]. Mindfulness-based training tailored to psychological well-being programs may also help reduce stress and overcome exercise barriers [16].

It should be noted that participating in an intervention programme may be time-consuming and can be challenging for participants due to their work schedules. Therefore, based on our systematic review findings, programmes should be designed to consider individual variations in needs and preferences. The delivery methods for these programmes need to be supported in the workplace and at an organisational level to ensure successful implementations for all participants. Additionally, the effectiveness of the programmes on participants’ well-being and physical activity levels should be tested in the short-term and long-term.

Future qualitative research is needed to identify optimal strategies to support physical activity in academics across various geographic settings. Robust assessment of programmes is essential, and this could include taking a developmental evaluation approach, using the RE-AIM framework to understand intervention reach, effectiveness, adoption, implementation and maintenance [84], and undertaking randomised controlled trials with large samples using a consistent physical activity measure. Future research should also explore the impact of physical activity on promoting goals in other areas for academics.

## Conclusions

This systematic review analysed 46 studies and identified low physical activity, long sedentary time in academics, and 42 factors associated with physical activity, categorised as sociodemographic, psychological, environmental, work-related, and health-related groups. Sedentary behaviours were associated with burnout, lower prospective mood, and lower energetic arousal. The barriers to physical activity included time constraints, distance to exercise facilities, financial costs, and work assignments. Seven interventions were identified as effectively promoting physical activity. Further research is needed to develop physical activity interventions in academics.

## Supplementary Information

Supplementary Material 1.

Supplementary Material 2.

Supplementary Material 3.

Supplementary Material 4.

Supplementary Material 5.

Supplementary Material 6.

Supplementary Material 7.

Supplementary Material 8.

## Data Availability

No datasets were generated or analysed during the current study.
