# Supplementary material for: Associated factors, barriers, and interventions to promote physical activity and reduce sedentary time in academics: a systematic review
Source: BMC Public Health. 2025 Aug 13;25:2753. doi: 10.1186/s12889-025-24092-2 (PMC12344990; doi:10.1186/s12889-025-24092-2)
Supplement: Supplementary file 1 — Supplementary Material 1. [file 12889_2025_24092_MOESM1_ESM.docx]

Supplementary Table 1: Search eligibility

|  | Inclusion | Exclusion |
| --- | --- | --- |
| Population | Teaching staff members, researchers, and supporting professionals (e.g., administrative workers) in academics | Employees working in non-higher educations. |
| Exposure | Universities, higher education, institutions | Non-universities, non-higher education, non-institutions |
| Outcomes | - Physical activity or sedentary behaviours as a primary outcome - Physical activity-related outcomes (e.g., pain, fitness) - Sedentary-related outcomes (e.g., pain) - Factors associated with physical activity and sedentary behaviours from three aspects: demographic, environmental and workplace - Barriers to physical activity - Effectiveness of interventions to improve physical activity and reduce sedentary time | - Non-discussion of factors associated with physical activity and sedentary behaviours from any of the three aspects: demographic, environmental or workplace - Non-discussion regarding the effectiveness of interventions to improve physical activity and reduce sedentary time |
| Type of studies | Quantitative, including experimental and observational studies | Non-quantitative studies |
| Publication | - English language of publication with full-text - Published between the inception and April 2024 | - Summaries, commentaries, reviews, case studies, qualitative studies, quantitative studies - Unable to obtain full-text articles - Non-English articles |
