# Supplementary material for: Associated factors, barriers, and interventions to promote physical activity and reduce sedentary time in academics: a systematic review
Source: BMC Public Health. 2025 Aug 13;25:2753. doi: 10.1186/s12889-025-24092-2 (PMC12344990; doi:10.1186/s12889-025-24092-2)
Supplement: Supplementary file 2 — Supplementary Material 2. [file 12889_2025_24092_MOESM2_ESM.docx]

Supplementary Table 3. Summary of physical activity measures from 46 studies

| Physical activity measure | Study | Number of studies |
| --- | --- | --- |
| International Physical Activity Questionnaire (IPAQ) | Almhdawi 2021; Cruz-Ausejo 2023; Demuth 2019; Kwiecień‑Jaguś 2021; Leininger 2015; Lopez-Olivares 2021; Shahlaee 2022; Yildiz 2023; Howie 2021; Opdenacker 2008 | 10 |
| Self-Developed Questionnaire | Diallo 2019; Freitas 2020; Galof 2021; Khubchandani 2009; Motevalli 2023; Omondi 2007; Pérussee-Lachance 2010; Redondo-Flórez 2020; Schmelling 1985; Soares 2019; Terzano 2011; Whipple 2008; Yorulmaz 2022; Zenbaba 2022 | 14 |
| Brief Questionnaire for Measuring Standard Physical Activity in Epidemiological Studies | Dias 2017 | 1 |
| Occupational Sitting and Physical Activity Questionnaire (OSPAQ) | Fountaine 2014; Headley 2018; Jones 2023; Wilkerson 2019 | 4 |
| Accelerometer | Giurgiu 2019; Headley 2018; Hudgins 2024; Brett 2017; Haines 2007; Howie 2021 | 6 |
| Global Physical Activity Questionnaire (GPAQ) | Hariyanto 2023 | 1 |
| Godin Leisure-Time Exercise Questionnaire (GLTEQ) | Hu 2021; Kirk 2012; Higham 2023 | 3 |
| A questionnaire physical discomfort of staff (ACSM) | Mohammadi 2016 | 1 |
| Dutch Musculoskeletal Questionnaire (DMQ) | Mohan 2015 | 1 |
| Ricci-Gagnon questionnaire | Moueleu Ngalagou 2019 | 1 |
| Godin-Shephard Leisure-Time Physical Activity Questionnaire (GSLTPAQ) | ÖZcan 2021 | 1 |
| International Physical Activity Questionnaire Short Form (IPAQ-SF) | Özdinç 2019 | 1 |
| Health Promotion Lifestyle Profile (HPLP) | Pirincci 2008 | 1 |
| The short questionnaire for the measurement of habitual physical activity in epidemiological studies | Sobhanian 2020 | 1 |
| Fitness test and psycho-behavioural inventory | Brinthaupt 2010 | 1 |
| Exercise and barriers to self-efficacy | Dawson 2008 | 1 |

Note: Howie 2021 used IPAQ and an accelerometer to measure PA levels; Headley 2021 used OSPAQ and an accelerometer to measure sedentary time.
