# Supplementary figures and images for: Associated factors, barriers, and interventions to promote physical activity and reduce sedentary time in academics: a systematic review
Source: BMC Public Health. 2025 Aug 13;25:2753. doi: 10.1186/s12889-025-24092-2 (PMC12344990; doi:10.1186/s12889-025-24092-2)

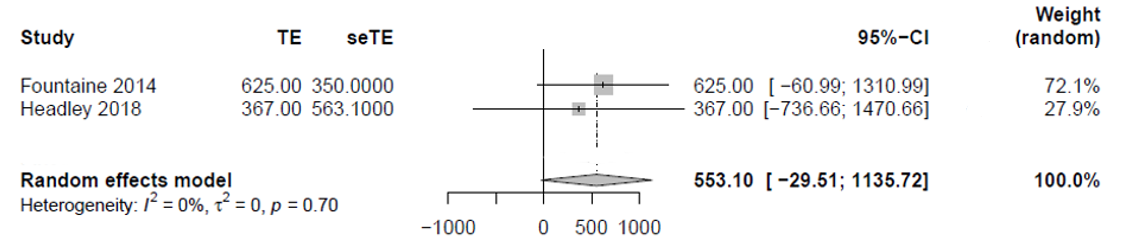


Supplementary Figure 1. A meta-analysis of sedentary time per workday from two studies

Supplement: Supplementary file 3 — Supplementary Material 3. [file 12889_2025_24092_MOESM3_ESM.docx]
