# Supplementary material for: Associated factors, barriers, and interventions to promote physical activity and reduce sedentary time in academics: a systematic review
Source: BMC Public Health. 2025 Aug 13;25:2753. doi: 10.1186/s12889-025-24092-2 (PMC12344990; doi:10.1186/s12889-025-24092-2)
Supplement: Supplementary file 4 — Supplementary Material 4. [file 12889_2025_24092_MOESM4_ESM.docx]

Supplementary Table 4. Factors associated with physical activity by study

| Factor | Study | Factor | Study |
| --- | --- | --- | --- |
| **Sociodemographic** |  | ***Environmental*** |  |
| Age | Demuth 2019 | Geographic Region | Motevalli 2023 |
| Sex | Demuth 2019 | Urban or rural areas | Motevalli 2023 |
|  | Hu 2021 | Transit Mode for Commuting to Work | Terzano 2011 |
|  | Redondo-Flórez 2020 | Inconvenience to Exercise | Whipple 2008 |
|  | Wilkerson 2019 | Weather | Whipple 2008 |
| Education Level | Wilkerson 2019 | COVID-19 Lockdown | Hudgins 2024  Shahlaee 2022 |
| Occupation | Wilkerson 2019 | ***Work-related*** |  |
| Job grade | Yildiz 2023 | Computer Usage Time | Yildiz 2023 |
| Smoking | Demuth 2019 | Lack of Free Time | Kirk 2012 |
| Alcohol Consumption | Kwiecień-Jaguś 2021 | Inconsistent Schedule | Kirk 2012 |
| **Psychological** |  | Heavy Work Demands | Kirk 2012 |
| Stress | Cruz-Ausejo 2023 | Burnout | Moueleu Ngalagou 2019 |
|  | Khubchandani 2009 |  | Yildiz 2023 |
|  | Soares 2019 | Less Time for Leisure | Moueleu Ngalagou 2019 |
| Negative Affect | Whipple 2008 | Work-Related Musculoskeletal Symptoms | Zenbaba 2022 |
| Hassle/Inconvenience to Exercise | Kirk 2012 | ***Health-related*** |  |
| Resistance to Exercise | Whipple 2008 | Pain | Özdinç 2019 |
| Exercise Self-Efficacy | Hu 2021 |  | Yorulmaz 2022 |
|  | Whipple 2008 |  | Diallo 2019 |
| Exercise Social Support | Hu 2021 |  | Galof 2021 |
| Job Satisfaction | Yildiz 2023 | Quality of Life | Yildiz 2023 |
| Positive Attitude | Kirk 2012 | Western Diet Pattern | Lopez-Olivares 2021 |
|  | Schmelling 1985 | Physical Fitness | Hu 2021 |
|  | Whipple 2008 |  | Kirk 2012 |
| Confidence | Whipple 2008 | Promoting Lifestyle Behaviours | Pirincci 2008 |
| Intention | Kirk 2012 | Poor Sleep Quality | Freitas 2020 |
|  | Schmelling 1985 | Ways of Spending Leisure Time in the Past | Demuth 2019 |
| Perceived Behaviour Control | Kirk 2012 | Health Status | Omondi 2007 |
| Taking Time from Obligations | Kirk 2012 |  | Kwiecień-Jaguś 2021 |
| Descriptive Norm | Kirk 2012 | Effect of COVID-19 on Quality of Life | ÖZcan 2021 |
