# Supplementary material for: Associated factors, barriers, and interventions to promote physical activity and reduce sedentary time in academics: a systematic review
Source: BMC Public Health. 2025 Aug 13;25:2753. doi: 10.1186/s12889-025-24092-2 (PMC12344990; doi:10.1186/s12889-025-24092-2)
Supplement: Supplementary file 5 — Supplementary Material 5. [file 12889_2025_24092_MOESM5_ESM.docx]

Supplementary Table 5. Summary of studies that reported barriers to physical activity

| **Study** | **Barriers identified in each study** |
| --- | --- |
| Khubchandani 2009 | - Time constraints (61.9%), - Work assignments (27.7%), - Cost of gym membership (24.6%), - Lack of parking (24.6%), and - Distance from the exercise facility (20.5%) |
| Leininger 2015 | - Time constraints (16.4%), - Resistance to using offered exercise programs (17.2%), - Living too far (6%), - Scheduling conflicts (11.4%), - Being unaware of the program (9.8%), - Not being interested in offerings (4.9%), and - Not being on campus often (3.3%). |
| Mohammadi 2016 | - Lack of motivation to exercise (1.29 [2.4]), - Many obligations and responsibilities of the family (1.26 [2.28]), - Busy and not having enough time to exercise (1.37 [3.39]), - Discouraging exercise by the university administration (1.37 [2.93]), - Lack attention to the development of sports culture in the workplace (1.29 [2.83]), - No instruction or directive that requires universities to provide support for sports (1.24 [3.08]), and - Lack of financial support from faculty and staff exercise (1.35 [3.13]) |
