# Supplementary material for: Associated factors, barriers, and interventions to promote physical activity and reduce sedentary time in academics: a systematic review
Source: BMC Public Health. 2025 Aug 13;25:2753. doi: 10.1186/s12889-025-24092-2 (PMC12344990; doi:10.1186/s12889-025-24092-2)
Supplement: Supplementary file 6 — Supplementary Material 6. [file 12889_2025_24092_MOESM6_ESM.docx]

| Research location | Study | Number of studies |
| --- | --- | --- |
| Australia | Giurgiu 2019; Higham 2023 | 2 |
| Austria | Motevalli 2023 | 1 |
| Belgium | Opdenacker 2008 | 1 |
| Brazil | Dias 2017; Freitas 2020; Soares 2019 | 3 |
| Cameroon | Moueleu Ngalagou 2019 | 1 |
| Canada | Kirk 2012; Pérussee-Lachance 2010; Dawson 2008 | 3 |
| China | Hu 2021 | 1 |
| Ethiopia | Zenbaba 2022 | 1 |
| Germany | Giurgiu 2019 | 1 |
| Indonesia | Hariyanto 2023 | 1 |
| Iran | Mohammadi 2016; Shahlaee 2022; Sobhanian 2020 | 3 |
| Jordan | Almhdawi 2021 | 1 |
| Kenya | Diallo 2019; Kenya | 2 |
| Malaysia | Mohan 2015 | 1 |
| Peru | Cruz-Ausejo 2023 | 1 |
| Poland | Demuth 2019, Kwiecień‑Jaguś 2021 | 2 |
| Slovenia | Galof 2021 | 1 |
| Spain | Lopez-Olivares 2021; Redondo-Flórez 2020 | 2 |
| Turkey | ÖZcan 2021; Özdinç 2019; Pirincci 2008; Yildiz 2023; Yorulmaz 2022 | 5 |
| UK | Brett 2017 | 1 |
| USA | Fountaine 2014; Headley 2018; Hudgins 2024; Jones 2023; Khubchandani 2009; Leininger 2015; Schmelling 1985; Terzano 2011; Whipple 2008; Wilkerson 2019; Brinthaupt 2010; Haines 2007; Howie 2021 | 13 |

Supplementary Table 2. Research locations of 46 studies
