# Supplementary material for: Associated factors, barriers, and interventions to promote physical activity and reduce sedentary time in academics: a systematic review
Source: BMC Public Health. 2025 Aug 13;25:2753. doi: 10.1186/s12889-025-24092-2 (PMC12344990; doi:10.1186/s12889-025-24092-2)

CINAHL Ultimate


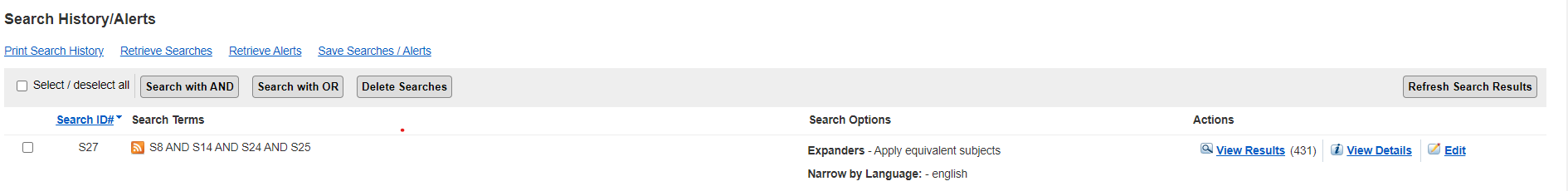


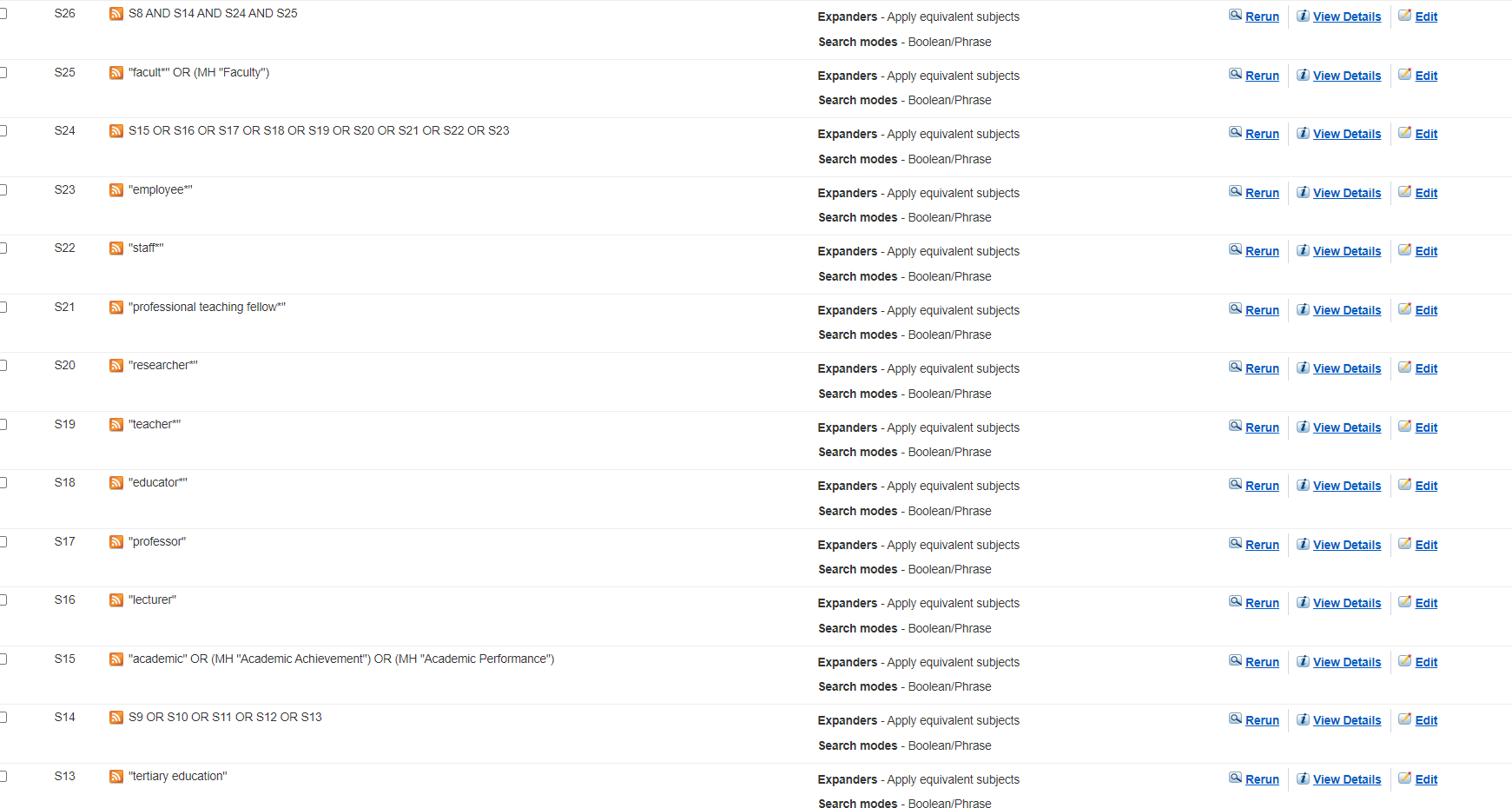


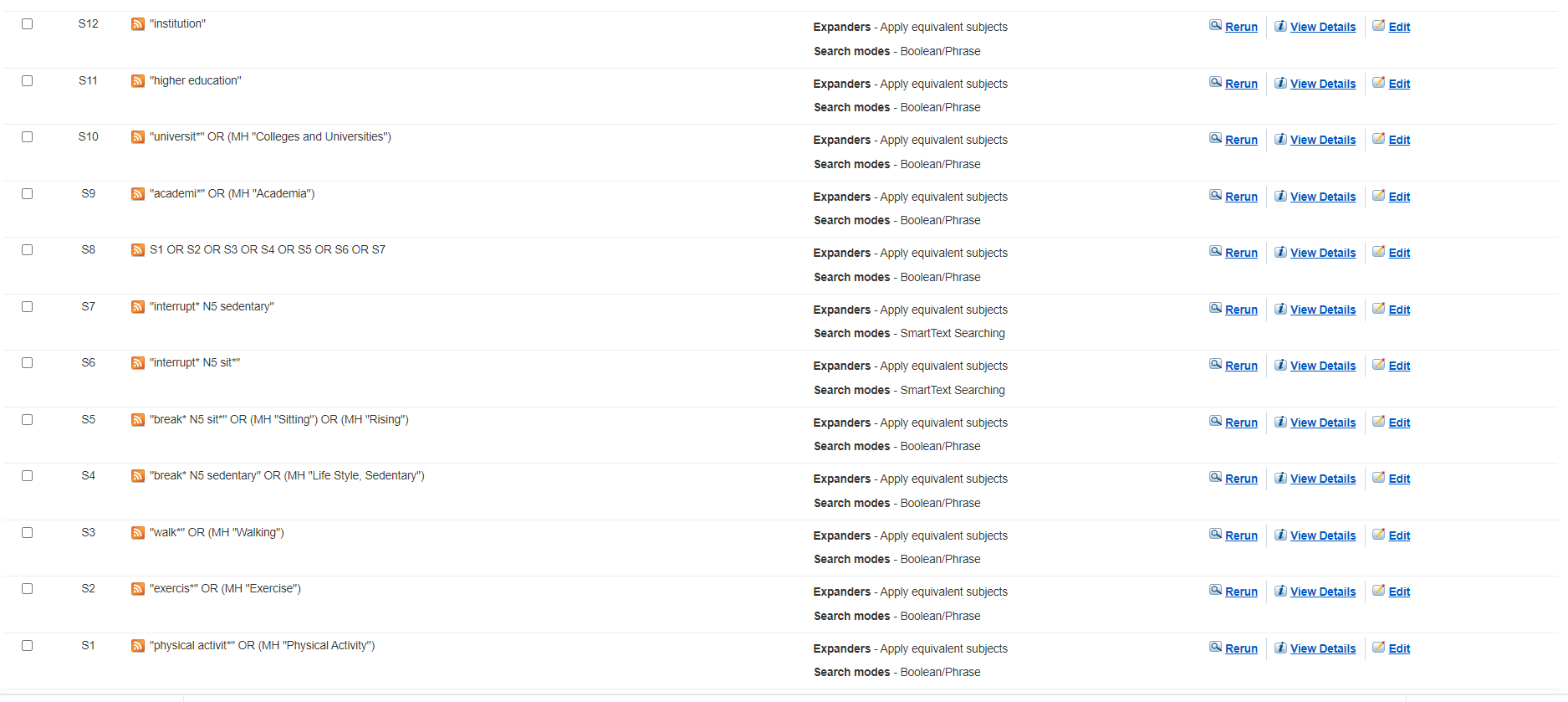


Cochrane


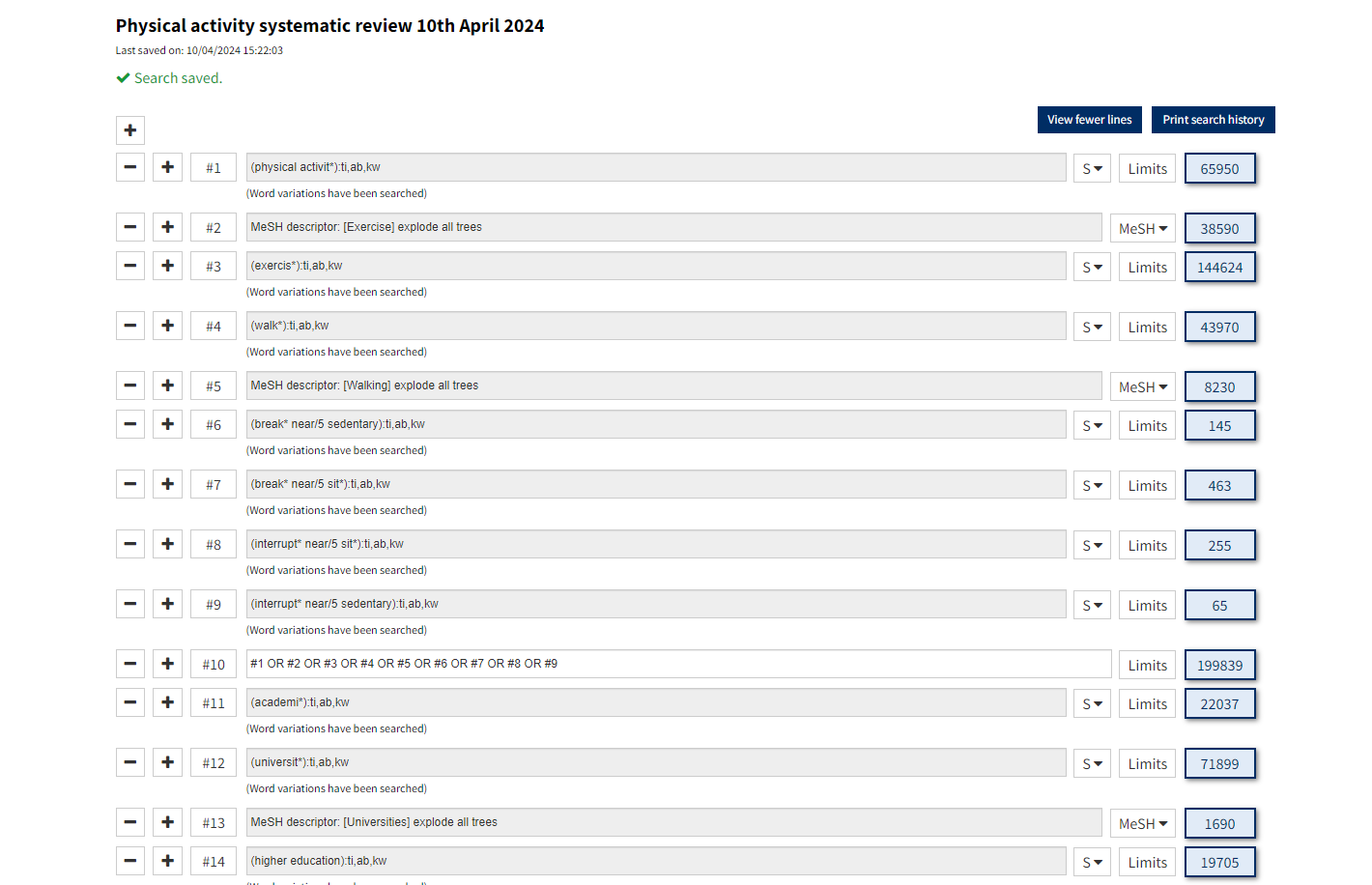


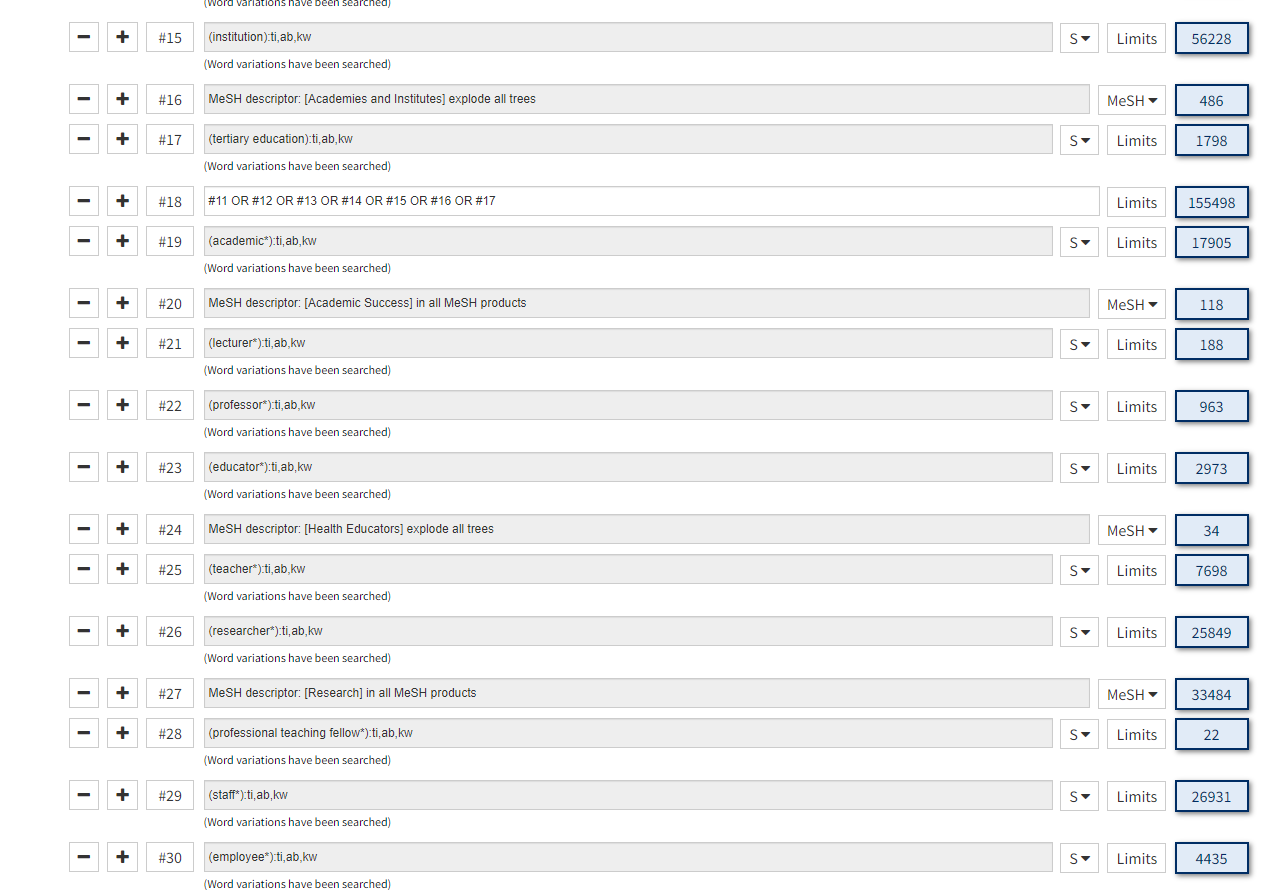


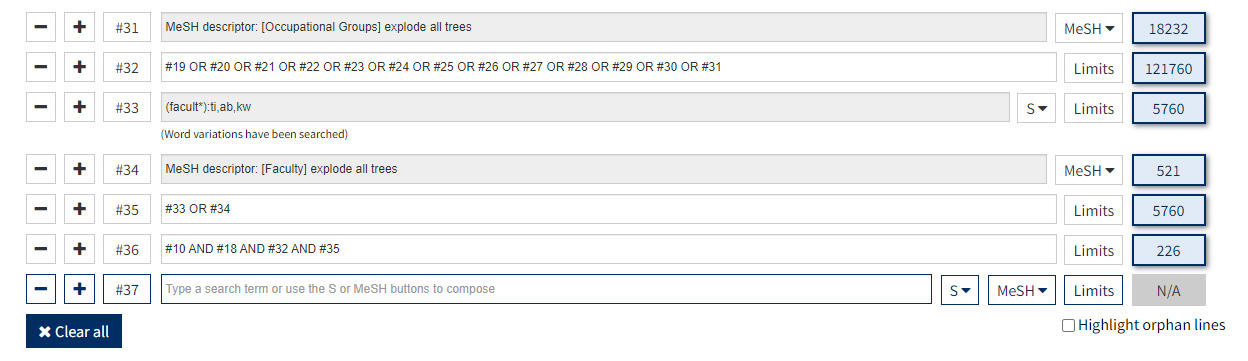


Medline (OVID)


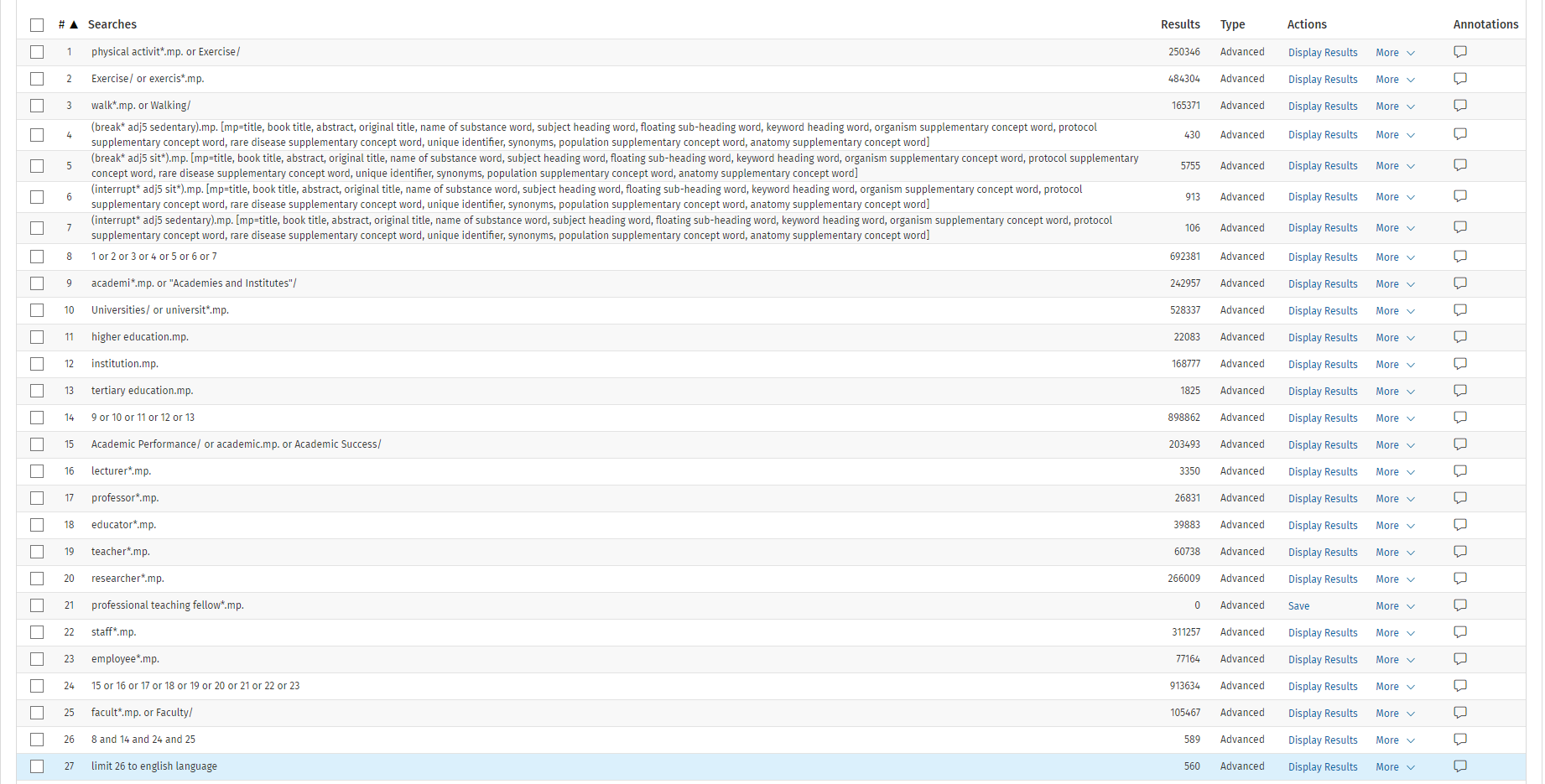


Scopus

( TITLE-ABS-KEY ( ( physical AND activit* ) OR exercis* OR walk* OR ( break* PRE/5 sedentary ) OR ( break* PRE/5 sit* ) OR ( interrupt* PRE/5 sit* ) OR ( interrupt* PRE/5 sedentary ) ) AND TITLE-ABS-KEY ( academi* OR universit* OR ( higher AND education ) OR institution OR ( tertiary AND education ) ) AND TITLE-ABS-KEY ( academic OR lecturer* OR professor* OR teacher* OR researcher* OR ( professional AND teaching AND fellow* ) OR staff* OR employee* ) AND TITLE-ABS-KEY ( facult* ) ) AND ( LIMIT-TO ( DOCTYPE , "ar" ) ) AND ( LIMIT-TO ( LANGUAGE , "English" ) )


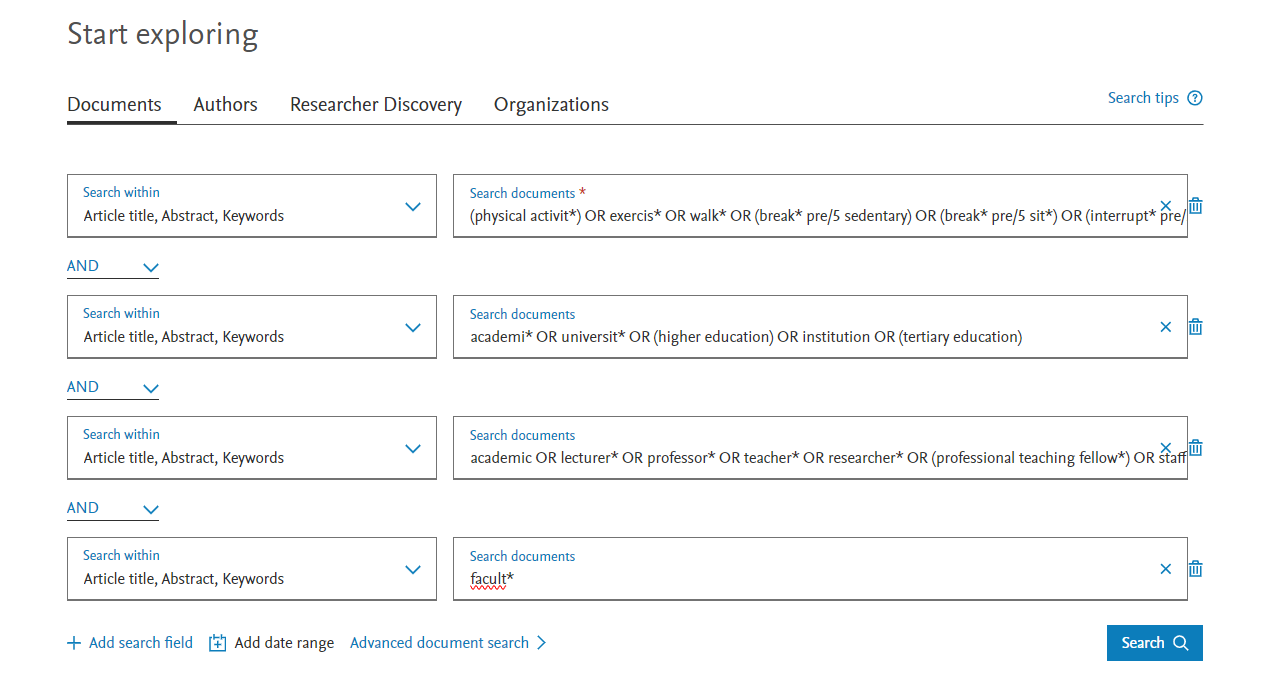


Searched on the 11^th^ April 2024

( TITLE-ABS-KEY ( "physical activit* " OR exercis* OR walk* OR ( break* PRE/5 sedentary ) OR ( break* PRE/5 sit* ) OR ( interrupt* PRE/5 sit* ) OR ( interrupt* PRE/5 sedentary ) ) AND TITLE-ABS-KEY ( academi* OR universit* OR "higher education" OR institution OR "tertiary education" ) AND TITLE-ABS-KEY ( academic OR lecturer* OR professor* OR teacher* OR researcher* OR "professional teaching fellow*" OR staff* OR employee* ) AND TITLE-ABS-KEY ( facult* ) ) AND ( LIMIT-TO ( DOCTYPE , "ar" ) ) AND ( LIMIT-TO ( LANGUAGE , "English" ) )v

SPORTDiscus


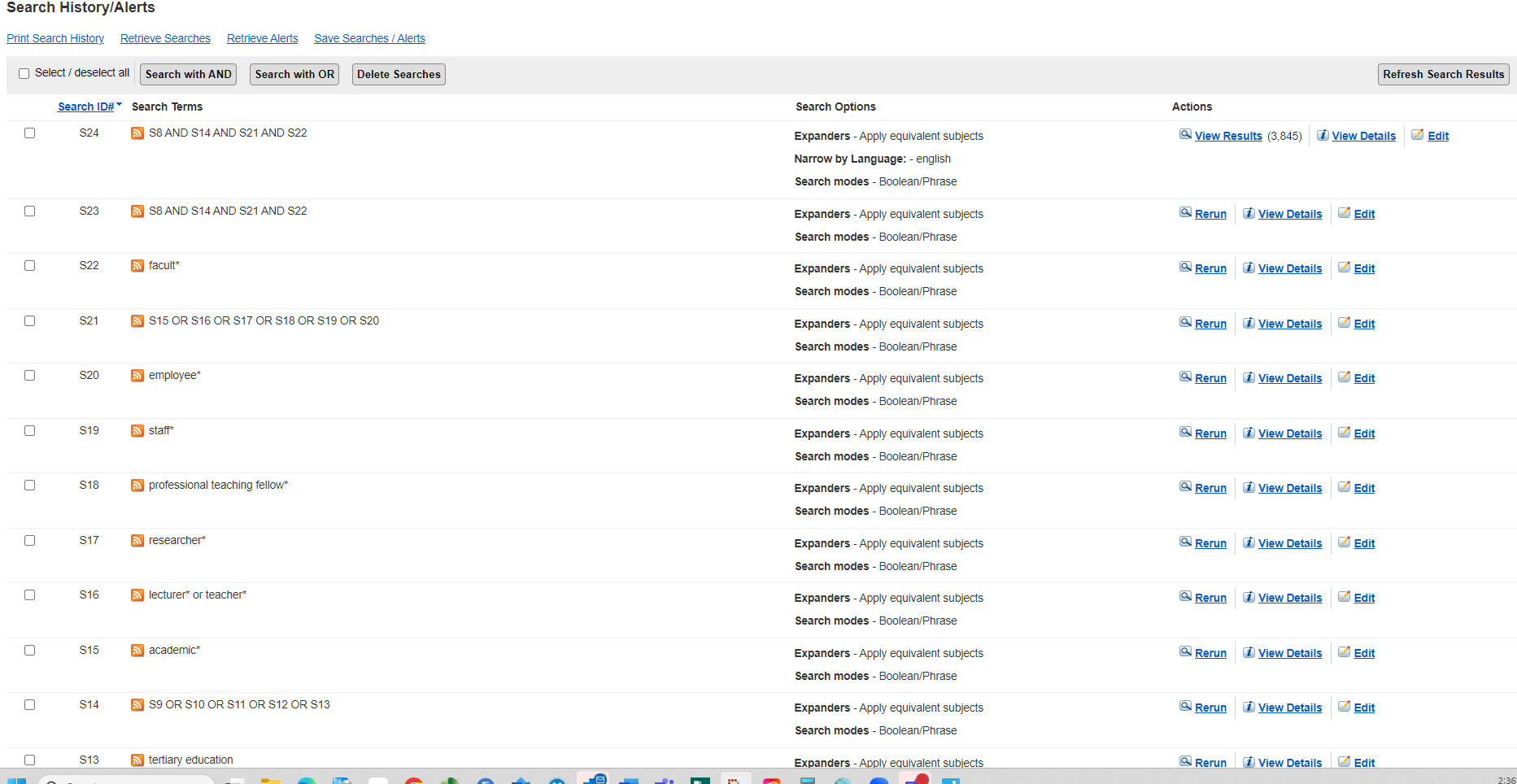


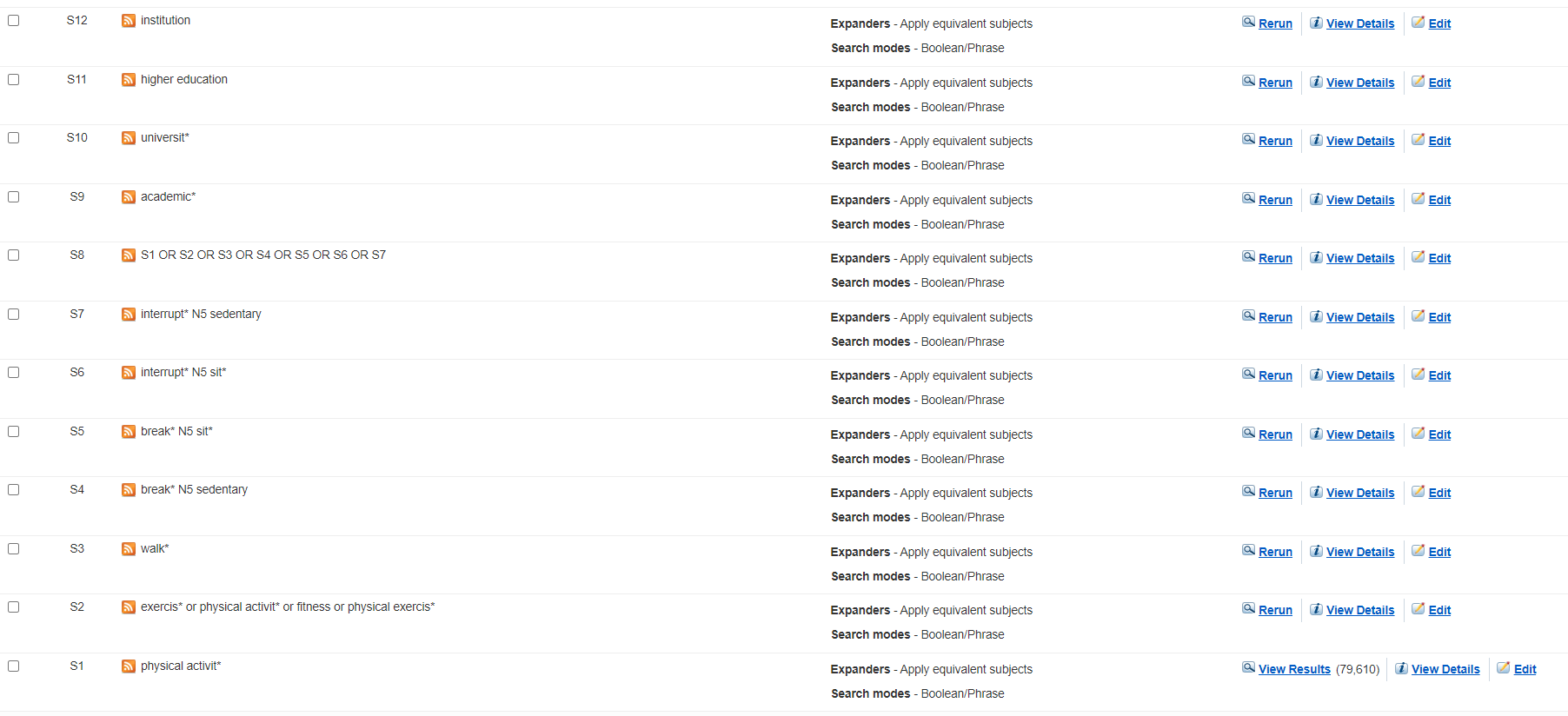

Supplement: Supplementary file 8 — Supplementary Material 8. [file 12889_2025_24092_MOESM8_ESM.docx]
